# Astrocyte Heterogeneity in the Adult Central Nervous System

**DOI:** 10.3389/fncel.2018.00401

**Published:** 2018-11-15

**Authors:** Sean J. Miller

**Affiliations:** Laboratory of Tony Wyss-Coray, Department of Neurology and Neurological Sciences, Stanford University School of Medicine, Stanford, CA, United States

**Keywords:** astrocyte, heterogeneity, diversity, adult, neurodegeneration, rodent, human

## Abstract

Astrocytes are the most abundant cell type in the central nervous system (CNS), providing critical roles in the overall maintenance and homeostasis. Over 100 years ago, Cajal first showed morphological depictions of different astrocyte populations. Surprisingly, to date astrocytes remain classified in two groups based on their morphological and neuroanatomical positioning. However, accumulating evidence over the past few years is showing that astrocytes are highly diverse throughout the CNS. Astrocyte heterogeneity is not surprisingly, as these cells interact with all other cells in the CNS. Like neurons, astrocytes may also have subpopulations that vary in their functionality. In this mini review, we will explore some of the recent evidence in the adult CNS of astrocyte diversity. First, we will review the very little literature on healthy adult astroglia heterogeneity, followed by the identification of different subpopulations in disease states and how this varies between human and mouse. Exploring this new area of neuroscience will hopefully provide researchers with a new perspective on astrocytes and their heterogeneity throughout the CNS.

## Introduction

In the adult central nervous system (CNS) there are many cell types including but not limited to: neurons, oligodendrocytes, microglia and astroglia. Each cell type plays essential roles in maintaining the CNS environment. However, the functionality of these cell types is highly diverse and dependent on countless factors, including their neuroanatomical localization (Krencik et al., 2011). Over the past several decades, researchers have identified a high degree of diversity within each of these cell types, and they have also learned much about how functions of these cell types are altered during disease pathogenesis (Miller et al., 2017). However, there still remain major gaps in our understanding about the identification and overall characterization of different astroglia populations. Of particular interest is how do these subpopulations regulate their local niche. Further, most studies to date have been performed in the developing CNS, providing little knowledge about cell type diversity in the mature adult CNS (Tsai et al., 2012; Molofsky et al., 2013).

Astroglia (also referred to as astrocytes) are the most abundant cell type in the adult CNS, where they play vital roles in the maintenance and homeostasis. Astroglia perform a vast array of functions including, but not limited to: ion buffering, neurotransmitter recycling, blood-brain-barrier maintenance, cholesterol synthesis, immune signaling, gliotransmitter release, neurotrophin secretion and regulation of neuronal synaptogenesis and elimination (Chen et al., 2006; Phatnani and Maniatis, 2015). However, these functions are performed in a diverse fashion by different subsets of astroglia (Zhang and Barres, 2010).

## Astroglia Subsets in Normal Health

Over 100 years ago Cajal first showed that human and rodent astroglia display a large number of different morphologies (John Lin et al., 2017; Dossi et al., 2018). In most literature since Cajal’s first observations, astroglia have been separated into two groups based on their gross neuroanatomical localization and morphological depictions: protoplasmic astroglia of the gray matter and fibrous astroglia of the white matter. However, over the past decade accumulating evidence has demonstrated that astroglia display a high degree of heterogeneity (John Lin et al., 2017; Liddelow et al., 2017). This heterogeneity is important for maintaining their microenvironment, where astroglia are constantly dealing with diverse cell types in different states in an ever-changing environment.

In the developing mouse spinal cord, it has been shown that fibrous astrocytes exhibit region-specific patterning (Molofsky et al., 2013). These regionally located astroglia populations perform different functions based on the neuronal populations they are associated with. Furthermore, depletion of region-specific astroglia in the neurodevelopment of the spinal cord resulted in the selected loss of neighboring neurons (Molofsky et al., 2014). However, this inadequately defines astroglia heterogeneity in the adult, especially protoplasmic astroglia.

In normal adult health, astroglia display physiological differences within the same brain regions (Höft et al., 2014). For instance, the gap junction coupling between astroglia have shown to vary largely between astroglia in the hippocampus (Matthias et al., 2003; Isokawa and McKhann, 2005). The different mouse astroglia populations have been coined “passive” and “complex” based on their expression of glutamate receptors and transporters and ion channels (Zhang and Barres, 2010). Passive astroglia have been shown to express glutamate transporters but not ionotropic glutamate receptors. In the contrary, complex astroglia express glutamate receptors but not transporters (Table 1, Supplementary Table S1). This heterogeneity in the same brain region shows that different astroglia populations perform different functions to maintain their environment.

**Table 1:** Illustrates astroglia diversity among common neurological disorders and in healthy states (Alzheimer’s disease (AD), Huntington’s disease (HD), amyotrophic lateral sclerosis (ALS), and Parkinson’s disease (PD)).

Identification/Neuroanatomical localization	Molecular signature	Notes/Phenotypes/Cellular signature
Neurodegeneration (HD, PD, AD, ALS)	Glt1^low^, Kir4.1^low^	Downregulation of essential ion and neurotransmitter channels and receptors; reactive; hypertrophied
Epileptic astrocytes	Kir4.1^low^, Glt1^low^, GS^low^	Hypertrophied, dis-localization of AQP4 (Coulter and Steinhäuser, 2015; Ohno et al., 2015)
A1 astrocytes	C3^high^, H2-T23^high^, Serping1^high^, H2-D1^high^, Ggta1^high^, Iigp1^high^, Gpp2^high^, Fbtln5^high^, Psmbb8^high^	Lethal to oligodendrocytes and neurons, phenotype initiated by microglia, strong correlation to neurodegeneration, decreased phagocytic ability, reactive phenotype (Liddelow et al., 2017); increased with aging (Clarke et al., 2018)
A2 astrocytes	Clcf1^high^, Tgm1^high^, Ptx3^high^, S100a10^high^, Sphk1^high^, Cd109^high^, Ptgs2^high^, Emp1^high^, Slc10a6^high^, Tm4sf1^high^, B3gnt5^high^, Cd14^high^, Stat3^high^	Promote neuronal survival (Liddelow et al., 2017); non-reactive phenotype (Clarke et al., 2018)
Olig2-lineage astrocytes	GFAP^low^, Hoxb4^high^, GLAST^low^	Originate from Olig2^+^ lineage (Tatsumi et al., 2018); neuroprotective post ischemeic brain injury (Jiang et al., 2013)

The cortex consists of several layers that have distinct neuronal populations. Thus, one hypothesis could be that these different neuronal populations in the cortical layers have cortical layer specific astroglia subpopulations as well. To examine Ca^2+^ activity between mouse cortical layers, Takata and Hirase (2008) performed multi-photon confocal imaging of astroglia in cortical layer I and layers II/III. During this investigation, they were able to show that Ca^2+^ activity was dramatically different between cortical layer I and layers II/III. Specifically, cortical layer I astroglia displayed twice as much Ca^2+^ activity compared to layers II/III (Takata and Hirase, 2008). Ca^2+^ signaling in astroglia promotes the release of gliotransmitters and signaling events, and the difference in Ca^2+^ activity could reflect the local needs of neighboring neurons and other cells in different cortical layers, further supporting the hypothesis that astroglia display great diversity between cortical layers (Lanjakornsiripan et al., 2018). However, human astroglia when compared to rodent astroglia propagate higher Ca^2+^ wave velocities and increased levels of Ca^2+^ signaling proteins (Dossi et al., 2018). Further research should focus on the secretion of cortical specific gliotransmitters.

Almost all *in vitro* experimentation on human and mouse astroglia has grouped astroglia into one cellular population, leaving varying results between research labs because astroglia are indeed heterogeneous, so unique populations will respond differently (Goursaud et al., 2009). Unfortunately, another caveat to conclusions drawn in pursuit of understanding astroglia biology is the lack of our understanding of adult astroglia heterogeneity in healthy states, since the vast majority of studies have been performed in diseased models (Rothstein et al., 1995; Tong et al., 2014).

One recent study addressing adult astroglia heterogeneity utilized fluorescence-assisted cell sorting (FACS) and immunological approaches (John Lin et al., 2017). John Lin et al. (2017) FACS astroglia from several different regions in the mouse CNS and discovered five different populations. The astroglia populations were categorized based on their affinity and expression to several antibodies. Furthermore, in this study, they showed that each astroglia population differentially regulated synaptogenesis on neurons and had varying contributions to human glioma and seizure onset (Table 1, Supplementary Table S1). These approaches are some of the very first to isolate different astroglia subsets in the healthy adult brain (John Lin et al., 2017).

A major function of astroglia is ion buffering. One major ion channel to buffer potassium ions is Kir4.1 (*Kcnj10*). Kir4.1 is an inward-rectified potassium channel that is critical for homeostatic potassium ion levels in the extracellular space (Farmer and Murai, 2017). In the normal adult CNS, Kir4.1 levels vary highly in gray matter astroglia (Olsen et al., 2007). The neocortex and hippocampus exhibit high heterogeneity in astroglial Kir4.1 levels (Table 1; Tang et al., 2009). However, in the cortex, cortical layers II/III and V display the highest levels of Kir4.1 immunoreactivity (Supplementary Table S1). Furthermore, this is an example of diversified functionality of different astroglia populations, where potassium ion buffering varies between neuroanatomical regions which may reflect the physiological needs of neighboring neurons. In several neurological paradigms, Kir4.1 levels are altered in certain astroglia subpopulations (discussed below; Kaiser et al., 2006; Ohno et al., 2015).

In normal aging, different brain regions exhibit transcriptional differences in astroglia. Clarke et al. (2018) performed RNA-sequencing (RNA-seq) on astroglia in the cortex, hippocampus, and striatum at five different aged time points. In this study, they show from RNA-seq and *in situ* hybridization that each brain region studied displayed a great level of astroglia heterogeneity. Furthermore, in aging the astroglia in these brain regions had significant changes in their transcriptional profile. The striatum and hippocampus, areas highly prone to neurodegeneration, displayed the highest level of diversity. Specifically, these subcortical regions upregulated reactive genes in astroglia to promote A1 astroglia (discussed below). The formation of A1 astroglia has been shown in many neurodegenerative disorders. Specifically, A1 astroglia have been shown to be toxic to both neurons and oligodendrocytes by releasing an unknown toxic factor. This study and several others further support astroglia heterogeneity between different brain regions but also shows different astroglia populations change their molecular profile in normal aging (Zhang and Barres, 2010; Clarke et al., 2018).

Other approaches for studying astroglia heterogeneity have come from disease models. In different neurodegenerative diseases, specific astroglia subsets downregulate key ion channels and neurotransmitter transporters (Rothstein et al., 1995; Tong et al., 2014). Understanding why protoplasmic astroglia in different regions respond differently to disease pathology is a major question in the field.

## Astroglia Subsets in Disease

Astroglia have been widely implicated a wide range of neurological disorders including models of epilepsy, brain tumors, mental retardation and neurodegeneration (Molofsky et al., 2012; Cabezas et al., 2014; Ohno et al., 2015; John Lin et al., 2017). However, understanding how different astroglia subsets promote or exacerbate disease pathogenesis is largely unexplored. Furthermore, different human and mouse astroglia populations respond differentially to neuronal insult, with some becoming hypertrophic, reactive, and senescent, while others begin to proliferate. Lastly, astroglia also respond to insult by the release of either inflammatory or anti-inflammatory molecules.

**Figure 1:**
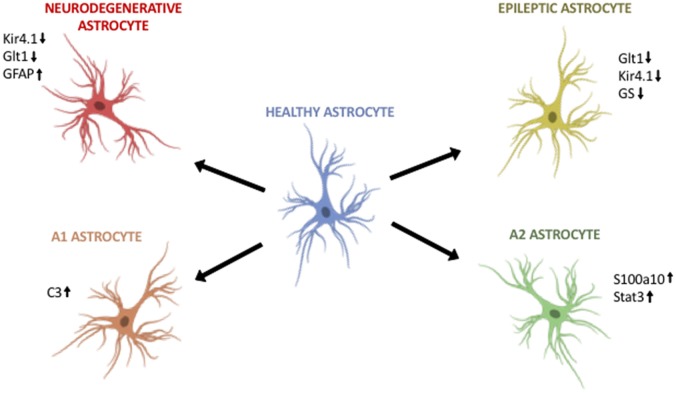
Astroglia diversity in the adult brain. (Top, left) Astroglia in neurodegenerative disorders differentially regulate Kir4.1, Glt1 and GFAP. (Top, right) Astroglia in epilepsy downregulate Glt1, Kir4.1 and glutamine synthetase (GS). (Bottom, left) A1 astroglia are neurotoxic and upregulate markers such as C3. (Bottom, right) A2 astroglia are neuroprotective and upregulate S100a10 and Stat3. Image modified from Cancer Research UK/Wikimedia Commons.

Epilepsy has long served as a neurological disorder with strong astroglia pathology. In epilepsy, human and mouse astrocytes downregulate essential neurotransmitter transporters and ion channels that are vital to maintain the proper physiological levels of neurotransmitters and ions. For instance, Kir4.1 and Glutamate transporter-1 (Glt1) are both significantly downregulated in epileptic human and rodent astroglia (Ohno et al., 2015; Dossi et al., 2018). This downregulation is thought to exacerbate disease pathology by allowing the accumulation of glutamate and potassium ions at the synaptic cleft, leaving neurons susceptible to these aberrant levels. An additional protein downregulated is the glutamate-converting enzyme, glutamine synthetase (GS; Figure 1, Table 1, Supplementary Table S1, Ohno et al., 2015). Lastly, it has been shown that in epilepsy, astroglia downregulate water channels such as perivascular-localized Aquaporin-4 (AQP4; Ohno et al., 2015). Not surprisingly, the combination of point mutations in the *Kir4.1* and *AQP4* genes is present in patients with antecedent febrile seizures and mesial temporal lobe epilepsy (Ohno et al., 2015; Dossi et al., 2018).

Recent evidence sheds light on two different reactive astroglia populations in the adult CNS: A1 and A2. Liddelow et al. (2017) demonstrated that A1 astrocytes can exacerbate disease pathogenesis and kill both neurons and oligodendrocytes. In contrast, A2 astrocytes appear to upregulate neurotrophic genes that promote neuronal survival. Further, activation of both A1 and A2 astroglia is result of cross-talk between activated microglia and astroglia in disease. When Liddelow et al. (2017) inhibit the inflammatory signaling molecules released by activated microglia, they are able to prevent A1 astroglia development. In addition, in multiple neurodegenerative diseases A1 astroglia are indeed present in mouse and human, particularly around areas of disease pathology (Figure 1, Table 1, Supplementary Table S1). *In vitro* studies showed that the astrocytic toxic effects were due to soluble factors released by A1 astroglia, although the exact identification of the factor, or factors, remains largely unknown even after more than a decade of research.

In Huntington’s disease (HD) research it is widely accepted that neurons in the striatum are hyperexcitable and degenerate. Tong et al. (2014) showed that astroglia in the striatum are also affected. One critical inward-rectifying potassium ion channel, Kir4.1, is highly downregulated in striatal astroglia (Figure 1, Table 1, Supplementary Table S1). In this study, the authors show that downregulation of Kir4.1 led to increased extracellular potassium. Using adeno-associated viruses (AAVs) to specifically target striatal astroglia and upregulate Kir4.1 levels resulted in improvement of striatal neuron survival, loss of neuronal hyperexcitability, and improvements in motor deficits. Therefore, strategies that target specific astroglia populations can restore neuronal deficits in neurodegenerative conditions (Tong et al., 2014).

Alzheimer’s disease (AD), the most common form of dementia, exhibits astroglia pathology even before neuronal death. In AD, human and rodent astroglia have aberrant calcium signaling, changes in reactivity, display vastly different gene profiles, changes in metabolism, upregulation of GFAP, and the release of GABA (Rodríguez-Arellano et al., 2016; Dossi et al., 2018). Patients with AD display elevated levels of GABA in their cerebrospinal fluid. In a recent study, the authors decreased the production and release of GABA from astrocytes in AD mice, and the treated AD mice had improvements in memory retention (Figure 1, Table 1, Supplementary Table S1, Jo et al., 2014). Moreover, AD patients’ astroglia have been shown to accumulate amyloid-β (Dossi et al., 2018). This demonstrates another neurodegenerative disorder that has a strong astroglia pathology and suggests approaches for the development of useful therapeutics.

Parkinson’s disease (PD) has recently shown to have aberrant astroglia in areas of pathology in both animal models and human post-mortem tissue. Chinta et al. (2018) recently showed that PD mutant astroglia exhibit senescence and the release of pro-inflammatory molecules which may exacerbate dopaminergic neurodegeneration in disease (Booth et al., 2017; Chinta et al., 2018). Furthermore, Chinta et al. (2018) showed that depletion of senescent-like astroglia leads to improved motor function and neuronal health. PD human and rodent astroglia have also been shown in other studies to become hypertrophied which results in a major change in gene expression, resulting in increased release of molecules such as IFNγ and TLR4 activation (Figure 1, Table 1, Supplementary Table S1, Booth et al., 2017).

Amyotrophic lateral sclerosis (ALS) has long served as an example of an astroglia disorder (Haidet-Phillips and Maragakis, 2015). In ALS human patients and their animal models, layer V cortical motor neurons and spinal cord motor neurons progressively degenerate, leading to severe and progressive motor impairments. These regions where pathology is observed exhibit downregulation of Glt1 in astroglia. This downregulation of Glt1 leaves excess glutamate in the extracellular space that can induce excito-toxic effects on motor neurons. This same subpopulation of astroglia also downregulate Kir4.1, which could serve to exacerbate these hyperexcitable deficits (Kaiser et al., 2006). Transcriptomic data shows that astroglia in an ALS mouse model alter greatly their transcriptome during disease progression (Miller et al., 2017). In *in vitro* models of ALS, human and rodent astroglia release neurotoxic factors to motor neurons, rendering them susceptible to degeneration (Figure 1, Table 1, Supplementary Table S1, Phatnani et al., 2013). The origin of these astroglia-specific changes is of great interest to neuroscience researchers but they still remain elusive.

Among all of the neurodegenerative diseases, its consistent that Kir4.1, Glt1 and GFAP are all differentially regulated. The downregulation of Kir4.1 and Glt1 could lead to accumulations of potassium ions and glutamate, respectively. This downregulation could then alter the excitability of neurons, such has been shown in HD rodent models. Therefore, potential therapeutics that selectively target these astroglia subpopulations may be extremely useful. Lastly, the upregulation of GFAP and the hypertrophied phenotype of astroglia in disease is also interesting. As it has been shown that hypertrophied astroglia release pro-inflammatory and toxic molecules such as A1 astroglia. This combination of an inflamed environment and excess ions and neurotransmitters may be a prominent driver of neurodegeneration. Further studies should focus on restoring Kir4.1 and Glt1 levels, in addition to altering the phenotype of an A1 toxic astroglia into neuroprotective A2 astroglia.

## Conclusion

This mini review has illustrated that astroglia are not homogeneous but rather very diverse in normal adult health. Furthermore, these studies also demonstrate that specific subpopulations of astroglia are strongly affected in neurological disorders. To add to the complexity of this phenomenon, astroglia are constantly confronting changing environments, from neural circuit regulation to response to neuronal insult. Further knowledge on astrocyte subpopulations may serve as a potential therapeutic target for a wide range of neurological disorders. However, with the expanding studies being performed to further understand cellular heterogeneity in the CNS, gaining that knowledge is not out of reach.

## Author Contributions

SM prepared and wrote the entire manuscript.

## Conflict of Interest Statement

The author declares that the research was conducted in the absence of any commercial or financial relationships that could be construed as a potential conflict of interest.
